# Use of imiquimod topical therapy for management of malignant melanoma positive margins

**DOI:** 10.3389/fonc.2025.1666463

**Published:** 2025-10-29

**Authors:** Katelyn Lewis, Sara Islam, David P. Alper, Martin J. Carney, Jennifer Choi, Jonathan Leventhal, Stephan Ariyan, Raymond Baumann, Kelly Olino, James Clune

**Affiliations:** ^1^ Division of Plastic and Reconstructive Surgery, Department of Surgery, Yale School of Medicine, New Haven, CT, United States; ^2^ Department of Plastic and Reconstructive Surgery, University of Mississippi, Jackson, MS, United States; ^3^ Division of Plastic and Reconstructive Surgery, Department of Surgery, University of Rochester, Rochester, NY, United States; ^4^ Department of Dermatology, Northwestern University Feinberg School of Medicine, Chicago, IL, United States; ^5^ Department of Dermatology, Yale School of Medicine, New Haven, CT, United States; ^6^ Yale School of Medicine Department of Pharmacology, Database Management, Yale University School of Medicine, New Haven, CT, United States; ^7^ Division of Surgical Oncology, Department of Surgery, Yale University School of Medicine, New Haven, CT, United States

**Keywords:** melanoma “*in situ*”, melanoma, imiquimod, margins, skin cancer

## Abstract

**Introduction:**

The treatment of positive margins following primary resection of cutaneous melanoma remains controversial with the current standard of care being repeat surgical resection. 5% topical imiquimod therapy has been proposed as an alternative treatment for positive *in-situ* surgical margins. This study revisits the use of 5% topical imiquimod therapy as a method of clearing positive surgical margins following primary excision of cutaneous melanoma.

**Methods:**

A retrospective chart review of all Yale Melanoma Registry Database patients with positive melanoma *in situ* margins after excision of primary melanoma between January 2008 and December 2021 was conducted. Patients were included if they received 5% topical imiquimod therapy for treatment of positive melanoma *in situ* surgical margins. Demographics, treatment duration, treatment response, complications, follow-up time, and associated costs were recorded.

**Results:**

A total of 69 patients with positive margins post wide local excisions were treated with topical imiquimod therapy. 38 of these patients had histological diagnosis of melanoma at primary excision while the remainder, 31, had MIS. Five patients were excluded due to follow-up with an outside dermatologist, leaving 64 patients in the final cohort. Fifty-two percent of patients were female with a median age of 73 years old in the entire cohort of patients. Treatment duration with imiquimod ranged from four to twelve weeks of therapy with a median duration of 12 weeks. Clinical response rate after final biopsies was 84% over an average follow-up duration of 36 months. Six patients (8.7%) had recurrences in follow-up after negative scouting biopsies following treatment with an average follow-up duration of 1086 days or approximately 36 months.

**Conclusion:**

The use of 5% topical imiquimod therapy is a safe, cost-effective, and reasonable alternative approach in the management of positive surgical margins. The degree of inflammation around the site of disease may be used as a reliable predictor of outcome.

## Introduction

The incidence rates of malignant melanoma have been increasing over the past decade (1–4). Last year, it was estimated that over 200,000 melanomas were diagnosed in the United States (5). The current standard of care remains to be wide local excision with margins dependent on the depth or invasiveness of the melanoma, ranging from 5mm for *in-situ* to 2cm for greater than 1.0mm invasion (6). The goal of taking margins of normal appearing tissue is to ensure histopathologic disease control by securing negative, disease-free margins.

Despite taking adequate tissue margins, securing histologic margins that are disease-free continues to be a challenge that practitioners must face. The rates of positive margins have been reported in the literature to be as high as 15-25% (2, 7–11). Risks of positive margins on final surgical pathology of melanoma increase in cosmetically sensitive areas, anatomic regions such as the head/neck, MIS histological subtype, presence of regression, and increasing age of the patients (10, 12).

Management of positive margins remains a subject of debate. In some instances, re-excision of residual melanoma may suffice; however, this subjects the patient to an additional operation and potential for further disfigurement. Topical immunotherapy with imiquimod has become increasingly popular as a treatment adjuvant for melanoma *in situ*. We previously reported our successful experience with imiquimod for the topical treatment of positive histologic margins after definitive surgical resection of melanoma in a 22 patient cohort (13). The National Comprehensive Cancer Network’s Clinical Practice Guidelines for melanoma now include the recommendation that imiquimod be considered as a treatment option for selected patients with positive MIS margins after optimal surgery (14). Imiquimod is an immunomodulator that works by inducing a TH1 immune response, thereby leading to infiltration of cytotoxic T-cells that result in apoptosis of melanoma cells (15).

Few studies have expanded on the management of melanoma positive margins using topical immunotherapy. Ellis et al.’s review of the literature described a plethora of encouraging data through case reports, case series, and open-label studies; however, they highlighted the need for more comprehensive studies to develop reliable and reproducible treatment regimens (16). In this present study, our objective was to provide additional detailed assessment of results, complications, and patient outcomes of medical management of positive margins with 5% topical imiquimod therapy in a larger group of patients with *in-situ* and invasive melanoma.

## Methods

After receiving institutional review board approval, a retrospective chart review of all Yale Melanoma Registry Database patients with positive margins after excision of melanoma *in situ* or primary melanoma between January 2008 and December 2021 was conducted. Subjects were identified based on Current Procedural Terminology codes and included if they had ever had a melanoma excision with subsequent positive margins. Patients with acral lentiginous melanoma were excluded as we do not offer Imiquimod routinely to these patients. Patients were found to have varying thicknesses of melanoma through histopathologic analysis of surgical specimens. Patients with residual disease at margins after definitive surgical resection were offered a course of topical application of 5% Imiquimod or re-excision. Those who opted for treatment with Imiquimod were included in our investigation. Patients were excluded if their charts were incomplete or were lost to follow-up.

Patients began imiquimod therapy, typically starting at a frequency of five applications per week. The patients were monitored closely with regular follow-up, typically at four-week intervals. During each visit, the treatment region was gradually tailored based upon the patient’s clinical response (labeled as no response, mild, moderate, or exuberant response). A goal of 12 weeks of therapy was made. Posttreatment histological scouting biopsies were performed at 6–8 weeks following the last application of topical 5% imiquimod cream.

## Results

A total of 69 patients met initial inclusion criteria. Of these patients, five were lost to follow-up, leaving 64 patients in our final cohort. Patient demographics and details of primary lesions are described in **Table 1**. Thirty-three patients in the cohort were male and 36 were female. There was a median age of 73 years old at the initial excision of melanoma. At primary excision, 38 patients had the histological diagnosis of melanoma *in situ* (MIS) while the remainder (31) had invasive melanoma. Average follow-up time was 1086 days or 36 months. All 69 patients had MIS at the margin, and all affected margins were peripheral in location. No patients had invasive melanoma at the margin.

**Table 1:** Patient and operative related characteristics of the overall study population.

Variable	Total (n=69)		
Gender	MIS	Melanoma	Total
Female	23 (33.33%)	15 (21.87%)	52.17%
Male	15 (21.87%)	16 (23.19%)	47.83%
Age			70.6
Primary Team			
Plastic Surgery	29 (42%)	28 (40.6%	82.61%
Other	9 (13%)	3 (4.3%)	17.39%
Follow-Up Time (days)	1000	1213	1086
Involved Margin			
Peripheral	26 (100%)	21 (100%)	
Deep	0	0	
Reconstruction Time Frame			
Immediate	22 (43.1%)	17 (33.33%)	76.50%
Delayed	3 (5.90%)	10 (19.60%)	25.50%
Both	1 (1.90%)		1.90%
Management			
Primary Closure	6 (11.8%)	2 (3.90%)	15.70%
Secondary Closure	3 (5.09%)	0	5.90%
FTSG	0	3 (5.90%)	5.90%
Local Flap	13 (25.50%)	15 (29.4%)	54.90%
Pedicle Flap	3 (5.90%)	5 (9.80%)	15.70%
Local Flap & FTSG	1 (1.90%)	0	1.90%
Location			
Upper Extremity	2 (3.90%)	1 (1.90%)	5.90%
Lower Extremity	4 (7.80%)	1 (1.90%)	9.80%
Torso	0	1 (1.90%)	1.90%
Head & Neck	32 (62.7%)	11 (21.6%)	86.30%
Cheek	8 (15.7%)	5 (9.80%)	25.50%
Nose	5 (9.80%)	4 (7.80%)	17.60%
Eyelid	7 (13.7%)	1 (1.90%)	15.70%
Forehead	6 (11.7%)	1 (1.90%)	13.60%
Ear	2 (3.90%)	0	3.90%
Lip	2 (3.90%)	0	3.90%
Scalp	0	2 (3.90%)	3.90%
Neck	1 (1.90%)	0	1.90%
Number of Excisions			
1	21 (41.20%)	20 (39.20%)	80.40%
2	5 (9.80%)	4 (7.80%)	17.60%
3	0	1 (1.90%)	1.90%
Melanoma Histology			
MIS	28 (54.90%)	0	54.90%
Malignant melanoma		6 (11.8%)	11.80%
Lentigo maligna		6 (11.8%)	11.80%
Superficial spreading		4 (7.8%)	7.80%
Desmoplastic		1 (1.9%)	1.90%
Spindle Cell		1 (1.9%)	1.90%
MIS & Superficial Spreading		2 (3.9%)	3.90%
Severe Atypia		1 (1.9%)	1.90%
Unknown		2 (3.9%)	3.90%

When examining cases of melanoma by the anatomical location (**Table 1**), 58 patients had a lesion on their head/neck, six patients had a lesion on their lower extremity, three patients had a lesion on their upper extremity, and two patients had a lesion on their torso. Thirty-seven patients had one excision performed prior to imiquimod treatment, nine patients had two excisions performed, one patient had three excisions performed, and 22 patients had incomplete surgical records to accurately record the number of initial excisions performed.

The treatment duration ranged from four to twelve weeks of therapy with a median duration of 12 weeks (**Tables 2**, **3**). Fifty-five patients had one course of therapy, eight patients had two courses of therapy, and one patient had three courses of therapy to achieve clear surgical margins. Another round of therapy was started if margins were still positive on scouting biopsies.

**Table 2:** Imiquimod treatment characteristics of the overall study population.

Variable	Total (n=42)		
Weeks of Imiquimod Treatment	MIS	Melanoma	Total
Average	11.5	11.9	11.7
Range	4-12		6 - 12
Mode	12	12	12
Rounds of Imiquimod Treatment			
1 Course	20 (47.6%)	15 (35.7%)	83.30%
2 Courses	1 (2.38%)	5 (11.90%)	14.30%
3 Courses	1 (2.40%)	0	2.40%
Unknown			21.40%

**Table 3:** Comparison of the severity of cutaneous reaction to imiquimod treatment versus post-treatment biopsy results.

Variable	Negative Biopsy (n=54)	Positive Biopsy (n=10)	P	Odds Ratio (95% CI)	Total (n=64)
None	1 (1.9%)	4 (40.0%)	Reference	Reference	5 (7.8%)
Mild	2 (3.7%)	3 (30.0%)	0.50	2.6 [0.16 - 45.14]	5 (7.8%)
Moderate	17 (31.5%)	3 (30.0%)	**0.01**	22.6 [1.84 - 279.38]	20 (31.3%)
Severe	34 (63.0%)	0 (0.0%)	**<0.001**	207.0 [7.28 - 5888.46]	34 (53.1%)

Bold values indicate statistical significance.

The response to imiquimod treatment was assessed by comparing the severity of the cutaneous reaction to imiquimod to the post-treatment biopsy results. On initial scouting biopsies after completion of the course of topical therapy, 54 patients had no evidence of melanoma on histopathologic examination (**Table 4**). Of these patients with negative initial scouting biopsies the degree of reactive inflammation to imiquimod varied. One patient had no reaction, two patients had a mild reaction, 11 patients had a moderate reaction, and 21 had a severe reaction. The remaining patients did not have their reaction recorded in the chart. The remaining 10 patients had positive margins on post-treatment biopsy results. Of these patients, three had no reaction to therapy, three had a mild reaction, and three had a moderate reaction. One patient did not have their reaction recorded. After additional rounds of therapy, scouting biopsies were negative and none of these patients experienced a recurrence. Minor complications were minimal in our cohort (**Table 4**). three patients had non-cutaneous side effects from Imiquimod, which included fatigue, flu-like symptoms, and acute depression. All patients’ side effects eventually resolved. Six patients (8.7%) in the cohort had recurrences after negative margins were achieved through treatment and three patients had metastatic disease. Of these patients with recurrences, two occurred in four years after original excision, one occurred in three years, and the remainder occurred one years following original wide local excision. Two of these patients had MIS on original histopathologic diagnosis while the remainder had melanoma primarily of the lentigo maligna subtype. Four patients had a moderate response to Imiquimod therapy, one had a severe, one mild, and one patient’s response was not documented.

**Table 4:** Complications of the overall study population.

Variable	Total (n=51)		
	MIS	Melanoma	Total
Recurrences	3 (5.9%)	2 (3.90%)	9.80%
Metastatic Disease	0	3 (5.90%)	5.90%
Superficial Wound Infection	0	1 (1.90%)	1.90%
Dehiscence	0	1 (1.90%)	1.90%
Non-cutaneous side effects from Imiquimod	0	2 (3.90%)	3.90%
Flap failure	0	0	0%

## Discussion

This study revisited and evaluated the use of 5% topical imiquimod therapy for treatment of positive melanoma margins following primary surgical excision. We hypothesized that a treatment duration of 12 weeks of imiquimod therapy, tailored based on clinical response and patient’s tolerance to treatment, would be sufficient to clear positive margins. We were successfully able to achieve an 84% initial clinical response rate in our cohort of 64 patients. Achieving negative margins can be challenging with narrower excisions at anatomical regions such as the head/neck (10). It is likely that smaller than necessary margins are taken near cosmetically sensitive areas such as the lip, eyelid, and face leading to an increase in persistently positive margins in these areas. Our data further supports the clinical use of topical imiquimod therapy as a nonsurgical alternative for the treatment of positive margins after primary melanoma excision. To our knowledge, this is the largest cohort of patients treated with imiquimod for management of positive margins in the literature to date.

At our institution, patients with primary melanoma who have positive margins for melanoma *in situ* are offered two treatment options: Imiquimod therapy or surgical excision. We ensure that patients are thoroughly informed about Imiquimod treatment, including its 12-week duration and the reliance on scouting biopsies to confirm treatment success. Following the completion of therapy, we perform six circumferential biopsies 6–8 weeks after completion of Imiquimod treatment. By this time, all the inflammation has resolved ensuring more accurate histopathologic evaluation of biopsy specimens.

Patients are counseled that, unlike surgical excision where all margins are examined, biopsies only allow us to confirm clearance within the sampled areas. It remains possible for malignant cells to persist between biopsy sites, which could lead to recurrence. For this reason, most patients opt for re-excision after the first experience with a positive margin, valuing its more definitive nature. However, if margins remain positive after a second excision, we again offer Imiquimod as a treatment option. At this stage, more patients tend to choose Imiquimod to evaluate its therapeutic effectiveness.

The use of imiquimod for treatment of MIS can be traced back to 2000 (16). Yet, there is still no standardized treatment protocol for frequency of applications and duration of therapy. Steinmann et al. reported a use of three applications/weekly for a total duration of 12 weeks to successfully clear cutaneous melanoma metastasis at the breast (17). Naylor et al.*’*s open label trial of imiquimod for treatment of 30 patients with lentigo maligna demonstrated a 93% clinical response rate following three months of daily therapy (18).

As mentioned by previous studies, the degree of clinical response may be used as a reliable predictor of outcome and therefore guide treatment duration (13, 19, 20). Ly et al.’s trial showed that there was a statistically significant association between visible association and histopathologic clearance (19). In our present series of 64 patients, five patients had no visible inflammatory response to imiquimod and four had mild inflammatory responses. Four out of the five patients with no visible inflammatory response had surveillance biopsies showing persistently positive surgical margins despite treatment for 12 weeks, and 3 out of the five patients with mild inflammatory responses had surveillance biopsies showing persistently positive surgical margins. Our data further supports the observation that more exuberant inflammatory reactions signal better clinical outcomes.

Topical treatment regimens for melanoma vary greatly across the literature, and there are numerous studies demonstrating the use of imiquimod with other surgical and nonsurgical therapies. These include imiquimod and 5-fluorouracil as dual therapy, imiquimod and tazarotene, and a combination of imiquimod, 5-Fluorouracil, and Tretinoin (21–23). Since 5-FU is an antimetabolite able to induce apoptosis of tumor cells and its effect are enhanced in the presence of some cytokines, INF-α, INF-γ and IL-12, this suggests a synergistic effect when used in combination with imiquimod (23). The use of a keratolytic agent like tazarotene is thought to increase the efficacy of imiquimod by enhancing drug penetration through the skin barrier (24). Treatment with a keratolytic agent can be administered concurrently with imiquimod or weeks prior to the start of therapy. And finally, a retrospective study of patients with cutaneous melanoma metastases demonstrated the use of intralesional IL-2 with topical imiquimod and retinoid cream as a promising therapeutic regimen (25).

Commonly reported side effects of topical imiquimod therapy include local erythema, scaling, edema, burning pain, and tenderness. These mild reactions typically dissipate after discontinuation of treatment. Systemic side effects are rare from topical application but have been reported in the literature. A recent case report described a patient who experienced debilitating fatigue after applying imiquimod to a single cutaneous lesion (26). One patient in our cohort complained of fatigue and diarrhea after the initiation of treatment. Her diarrhea resolved in two weeks; however, the fatigue persisted until the course of imiquimod was complete. Another patient in our cohort developed severe acute depression that did not resolve until cessation of treatment. The only other complication in our series was a patient who experienced flu-like symptoms after two weeks of therapy. The patient self-discontinued imiquimod and scouting biopsies were negative at a follow-up visit.

This study has several limitations that are common to all retrospective reviews. One significant limitation is the relatively small sample size of 64 patients, which limits the precision of risk estimates. Nevertheless, this study represents the largest cohort reported to date in the published literature. Another such limitation would be the interobserver variation in the histological diagnoses and evaluation for positive margins on surgical specimens, although all pathology was examined at the same institution. Secondly, this study had a median follow-up of around 36 months, and thus clinical recurrence could have been missed. However, we have previously reported that 81 percent of all melanoma recurrences were diagnosed by 36 months after the primary melanoma (27). In the MIS literature, a similar median time to recurrence of 36 months has been reported (28). A third limitation was the possibility of variation in physician’s grading of inflammatory response as none, mild, moderate, or severe as this was graded subjectively without inter-rater validation. Furthermore, given the retrospective nature of this review, there were not photographs available for all patients thus we could not standardize the grading of the inflammatory response.

Obtaining multiple physicians’ clinical grades or collecting photographs would have helped decrease variability and improve reproducibility of this study. While post-treatment scouting biopsies can be useful, they are not as reliable as re-excision of the entire surgical margin followed by comprehensive histologic evaluation. Scouting biopsies carry a risk of false negatives due to sampling error. To mitigate this, we obtain multiple histologic samples from the area surrounding the residual scar. Nevertheless, it’s important to recognize the limitations of this approach. As a result, all patients in our cohort are closely monitored for any signs of recurrence, with additional scouting biopsies performed when there is clinical concern.

## Conclusion

Imiquimod is a safe, cost-effective treatment of positive margins after initial resection of cutaneous melanoma. Patients with positive margins demonstrating MIS should consider Imiquimod as an adjunct or in some cases and alternative to further resection. Patients with more exuberant reactions demonstrate better clinical response to therapy.

## Data Availability

The raw data supporting the conclusions of this article will be made available by the authors, without undue reservation.
